# Biochemical validation of a second class of tetrahydrofolate riboswitches in bacteria

**DOI:** 10.1261/rna.071829.119

**Published:** 2019-09

**Authors:** Xi Chen, Gayan Mirihana Arachchilage, Ronald R. Breaker

**Affiliations:** 1 Department of Molecular Biophysics and Biochemistry, Yale University, New Haven, Connecticut 06520-8103, USA; 2 Howard Hughes Medical Institute, Yale University, New Haven, Connecticut 06520-8103, USA; 3 Department of Molecular, Cellular and Developmental Biology, Yale University, New Haven, Connecticut 06520-8103, USA

**Keywords:** aptamer, dihydrofolate, folate biosynthesis, gene regulation, noncoding RNA, THF

## Abstract

We previously reported a large collection of structured noncoding RNAs (ncRNAs) that includes many riboswitch candidates identified through comparative sequence analysis of bacterial intergenic regions. One of these candidates, initially named the “*folE* motif,” adopts a simple architecture commonly found upstream of *folE* genes. FolE enzymes catalyze the first enzyme in the de novo folate biosynthesis pathway. Herein, we demonstrate that *folE* motif RNAs selectively bind the enzyme cofactor tetrahydrofolate (THF) and several of its close derivatives. These aptamers, commonly found in Gram-negative bacteria, are distinct from aptamers of the previous validated THF riboswitch class found in Gram-positive bacteria. Our findings indicate that *folE* motif RNAs are aptamer domains for a second THF riboswitch class, named THF-II. The biochemical validation of THF-II riboswitches further highlights the ability of bacteria to utilize diverse RNA structures to sense universal enzyme cofactors that are predicted to be of ancient origin.

## INTRODUCTION

Riboswitches are structured RNA domains that alter gene expression in response to binding small molecule or elemental ion ligands (Roth and Breaker 2009; Breaker 2011; Serganov and Nudler 2013; Sherwood and Henkin 2016). More than 40 classes of riboswitches have been discovered to date, and most of these sense compounds that have been proposed to be of ancient RNA World origin (Breaker 2012; Nelson and Breaker 2017). Indeed, the most prevalent ligand types sensed by the known classes of riboswitches are enzyme cofactors, or their immediate precursors or byproducts (McCown et al. 2017). Recent reports describing the validation of additional riboswitch classes have added to this list. For example, distinct riboswitch classes have been uncovered for the enzyme cofactor *S*-adenosylmethionine (SAM) (Mirihana Arachchilage et al. 2018), and for the thiamin pyrophosphate precursor called 4-amino-2-methyl-5-hydroxymethylpyrimidine pyrophosphate (HMP-PP) (Atilho et al. 2019). Given this trend, it seems likely that additional riboswitch classes will be discovered that respond to enzyme cofactors and other nucleotide-derived ligands.

In the current report, we describe the validation of a second riboswitch class for the coenzyme tetrahydrofolate (THF), called THF-II. Members of this riboswitch class were initially named “*folE* motif” RNAs, and were reported (Weinberg et al. 2017) among a large collection of bacterial noncoding RNA domains that exhibited evidence for the presence of conserved sequences and secondary structures. Although the original consensus sequence and structural model indicated that the *folE* motif was less complex than most known riboswitch classes, its common association with genes coding for the FolE protein provided some support for the hypothesis that the motif represented a novel riboswitch class. FolE proteins are GTP cyclohydrolase enzymes that promote the first step in the pathway for the biosynthesis of folate.

Using comparative sequence analysis, we found that *folE* motif RNAs form a somewhat more complex secondary structure than was originally proposed. In addition, we use biochemical assays to demonstrate that *folE* motif RNA constructs, designed based on the revised consensus model, function as selective aptamers for THF and certain other folate derivatives, including dihydrofolate. These and other characteristics indicate that *folE* motif RNAs are components of riboswitches for THF and some of its related natural derivatives. Compared to the structural and functional characteristics of the previously reported THF riboswitch class (Ames et al. 2010; Trausch et al. 2011), the *folE* motif RNAs are distinct. Therefore, we recommend calling *folE* motif RNAs “THF-II” riboswitches, and renaming THF riboswitches as “THF-I.” Overall, our findings further reveal the structural and functional diversity of RNAs, and how modern riboswitches are extensively used by some bacterial species to sense key enzyme cofactors and to regulate fundamental metabolic processes.

## RESULTS AND DISCUSSION

### Updating the consensus model and gene associations for *folE* motif RNAs

**FIGURE 1.**
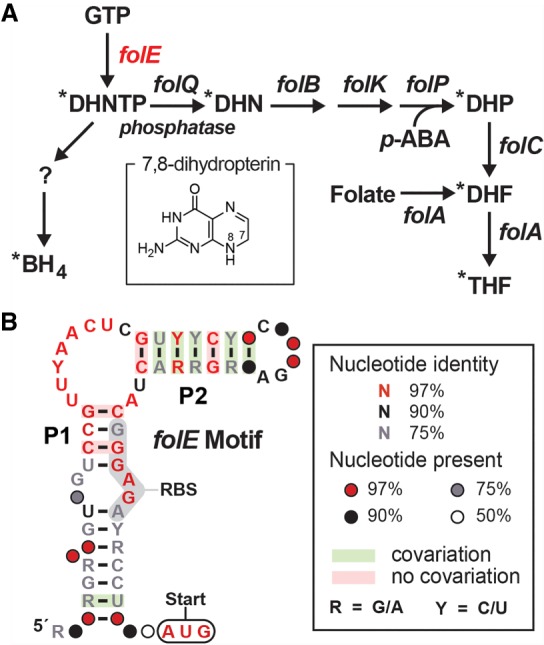
Gene association and consensus model for *folE* motif RNAs (THF-II riboswitches). (*A*) Typical tetrahydrofolate (THF) biosynthesis pathway in bacteria, and an additional branch for tetrahydrobiopterin (BH_4_) biosynthesis. Shared folate and biopterin biosynthesis intermediate: (DHNTP) dihydroneopterin 3′-triphosphate. Exclusive folate biosynthesis intermediates: (DHN) dihydroneopterin, (DHP) dihydroneopterin, (DHF) dihydrofolate. Exclusive BH_4_ intermediate: “?” represents PTP (6-pyruvoyl-5,6,7,8-tetrahydropterin) or another possible intermediate. All *folE* motif representatives are associated with the *folE* gene (red), which codes for the enzyme GTP cyclohydrolase I. Asterisks identify compounds carrying a 7,8-dihydropterin moiety (*inset*) as produced by the FolE enzyme, or its 5,6,7,8-tetrahydropterin derivative. (*B*) Updated consensus sequence and secondary structure model for the *folE* motif, based on 86 unique representatives. The predicted RBS (ribosomal binding site) is highlighted in gray, and the predicted AUG translation start site is circled.

The original consensus sequence and proposed structural model for *folE* motif RNAs was reported (Weinberg et al. 2017) as part of a collection of 224 structured noncoding RNA (ncRNA) candidates identified by comparative sequence analyses of bacterial intergenic regions (IGRs). Specifically, 51 representatives of the *folE* motif with distinct nucleotide sequences were identified, and many of these were located in the putative 5′ untranslated region (UTR) of a gene (*folE*) encoding GTP cyclohydrolase I. This enzyme catalyzes the first committed step in the de novo biosynthesis pathway for the enzyme cofactor folate or related compounds such as biopterin (Fig. 1A). The original consensus sequence and secondary structure model reported for the *folE* motif included numerous highly conserved nucleotides, but only a single predicted stem–loop structure (Weinberg et al. 2017). Regardless, given the strong sequence conservation and consistent gene association, we chose to further investigate the possibility that *folE* motif RNAs function as riboswitch aptamers.

Using comparative sequence analysis methods, we search for additional representatives of the *folE* motif within DNA databases including fully sequenced bacterial genomes and bacterial metagenomes (see Materials and Methods). This effort yielded an additional 35 representatives, to reach a total of 86 unique RNA sequences (Supplemental Data File), all present from species in the order Rhizobiales or in metagenomics DNA sequences. A revised and expanded consensus sequence and a secondary structure model were then generated based on this entire collection (Fig. 1B). This revised model includes two base-paired substructures, called P1 and P2, wherein the putative ribosome binding site (RBS) of the adjacent downstream gene is consistently part of the right shoulder of P1. This architecture suggests that the *folE* motif encompasses part of a riboswitch “expression platform” (Barrick and Breaker 2007; Breaker 2018), and that ligand binding might stabilize formation of an anti-RBS structure. If true, then the RNAs function as genetic “OFF” switches by blocking ribosome access to the adjacent AUG start codon, such that ligand binding prevents translation. Furthermore, the formation of the P1 stem restricts most of the conserved nucleotides to an asymmetric internal bulge joining the P1 and P2 stems. Riboswitch aptamers commonly use highly conserved nucleotides in the bulges of secondary structures to form ligand-binding pockets.

Given the various initial and newly recognized characteristics of these RNA representatives, we were prompted to experimentally investigate the *folE* motif as a candidate for a novel riboswitch class. To help inform the design of these experiments, we reexamined the gene associations that were originally reported for the motif (Weinberg et al. 2017). The genes located immediately downstream from the riboswitch candidate were annotated as *folE* (COG0302), TFold (cd00651), and GTP_cyclohydrol (cd00642) by the DNA database RefSeq version 63. On reinspection, these genes are now all annotated as *folE* in RefSeq version 80, which codes for GTP cyclohydrolase I. This observation strongly suggests that the *folE* motif is involved in sensing a molecule that incorporates a pterin-like moiety (Fig. 1A, inset), such as is carried by THF and its various precursors and natural derivatives.

Strangely, the gene immediately following *folE*, and perhaps occasionally in the same transcript, is always annotated as *hisI*. HisI proteins have been demonstrated to function as essential cyclohydrolase enzymes for the production of the amino acid histidine (Smith and Ames 1965; Sivaraman et al. 2005). Although this association with *folE* motif RNAs could have cast doubt on the hypothesis that the riboswitch ligand carries a pterin moiety, we find that this gene arrangement is common even in species of Rhizobiales that do not carry *folE* motif representatives. Furthermore, a connection between folate deficiency and *hisI* expression has been previously established in bacteria (Berberich et al. 1966). Therefore, we hypothesized that *folE* motif is a strong riboswitch candidate for THF or related intermediates in the folate biosynthesis pathway.

### RNA constructs carrying the *folE* motif consensus directly bind THF

**FIGURE 2.**
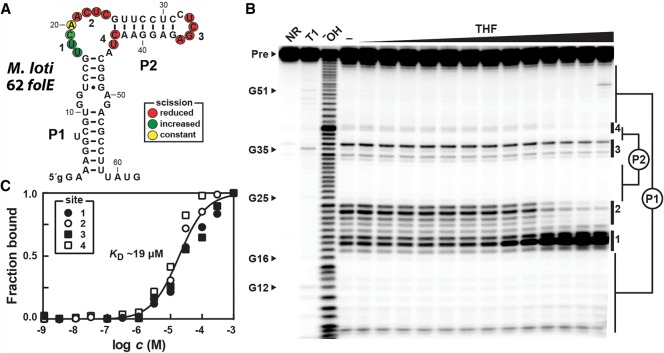
A representative *folE* motif RNA directly binds THF. (*A*) Sequence and secondary structure model of the 62 *folE* RNA construct based on the representative from *M. loti*. The lowercase letter on the 5′ terminus designates a G residue added to enhance in vitro transcription efficiency. Colored circles identify nucleotides whose phosphodiester linkages undergo scission during in-line probing reactions, wherein THF induces changes as indicated. Data are derived from the autoradiogram in *B*. (*B*) PAGE analysis of in-line probing reactions of 5′ ^32^P-labeled *M. loti* 62 *folE* RNA in the absence (−), or the presence of THF ranging in concentration from 1 nM to 1 mM. NR, T1, and OH^−^ indicate no reaction, partial digestion with T1 ribonuclease (cleaves after each G), and partial digestion under alkaline conditions (cleaves after every nucleotide), respectively. Bands corresponding to the precursor RNA (Pre) and several products of RNase T1 digestion (numbered as annotated in *A*) are indicated. Notable sites of the RNA that undergo modulation upon addition of THF are numbered 1 through 4. (*C*) Plot of fraction of RNA bound to ligand versus the logarithm of the concentration of THF, as determined based on quantification of band intensity changes at sites 1 through 4 in *B*. A line representing a theoretical 1:1 binding curve (Hill coefficient of 1) with a *K*_D_ of 19 µM is overlaid on the data points (*R*^2^ value of 0.97) for comparison.

To assess the ligand binding function of *folE* motif RNAs, we prepared a 62-nt RNA construct derived from the 5′ UTR of the *folE* gene of *Mesorhizobium loti*, named 62 *folE* (Fig. 2A). This representative also includes the predicted ribosome binding site (nucleotides 47–52) and the AUG start codon of the adjacent coding region (nucleotides 60–62). This construct was subjected to in-line probing (Fig. 2B), which reveals the locations of ligand-mediated changes in RNA structure through changes in the rate of spontaneous cleavage of their phosphodiester linkages (Soukup and Breaker 1999; Regulski and Breaker 2008).

In-line probing data reveal that the 62 *folE* RNA adopts the consensus secondary structure, and that portions of the RNA alter their structural flexibility in response to THF addition. Nucleotide positions that undergo a reduction in spontaneous cleavage reside in the P2 loop and in the bulge that links the P1 and P2 stems (Fig. 2A), suggesting that these nucleotides are at least partly responsible for ligand recognition and/or ligand-binding pocket formation. In addition, nucleotides 17–19 (site 1) exhibit increased cleavage in response to increasing ligand concentrations. We speculate that the linkages of these nucleotides might be held in an in-line orientation, or become structurally more flexible upon ligand binding.

To determine the binding affinity of THF-II aptamers, band intensities at sites 1–4 (Fig. 2B) were quantified and used to estimate the fraction of RNA bound to ligand (see Materials and Methods). A plot of the logarithm of the concentration of THF versus the fraction of RNA bound to ligand (Fig. 2C) is consistent with a 1:1 complex formation between RNA and ligand, although other explanations for the data are possible. The apparent dissociation constant (*K*_D_) for THF is ∼19 µM. Similarly, we examined the ability of another *folE* motif representative to bind THF. In-line probing assays using a 62-nt RNA from *Ochrobactrum intermedium* also yield data consistent with a 1:1 stoichiometry and an apparent *K*_D_ of ∼4 µM (Supplemental Fig. S1A).

**FIGURE 3.**
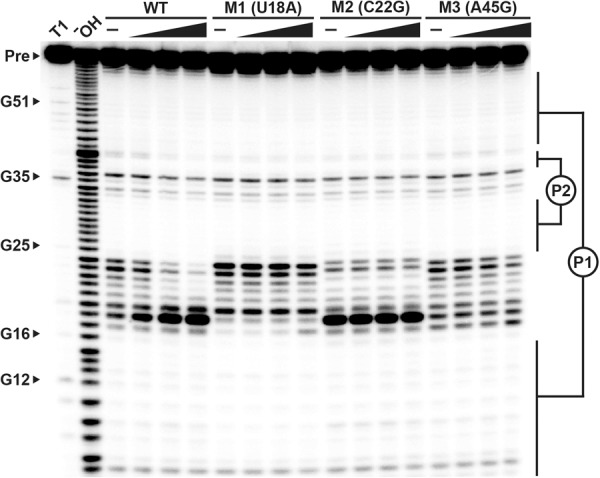
Conserved nucleotides are critical for THF binding by a representative *folE* motif RNA. The autoradiogram is the result of PAGE separation of in-line probing assay products for the wild-type (WT) *M. loti* 62 *folE* RNA and three mutant RNA constructs in the presence of 0 (–), 3, 30, and 300 µM THF (from *left* to *right*). Additional annotations are as described in the legend for Figure 2B. Positions of the mutations are based on the nucleotide numbering depicted in Figure 2A.

The importance of conserved nucleotides forming the *folE* motif consensus model was assessed by examining the effects of single mutations (Fig. 3, M1 through M3) on ligand binding. All three mutants tested fail to respond to THF even at concentrations as high as 300 µM. These results suggest that *folE* motif RNAs make specific interactions with THF, and that nucleotides in the highly conserved single-stranded regions are essential for ligand binding. Moreover, these findings support our hypothesis that *folE* RNAs function as aptamers for a distinct riboswitch class that can monitor the concentration of THF.

### The binding pocket of *folE* motif RNAs are distinct from those of the previously known THF riboswitch class

**FIGURE 4.**
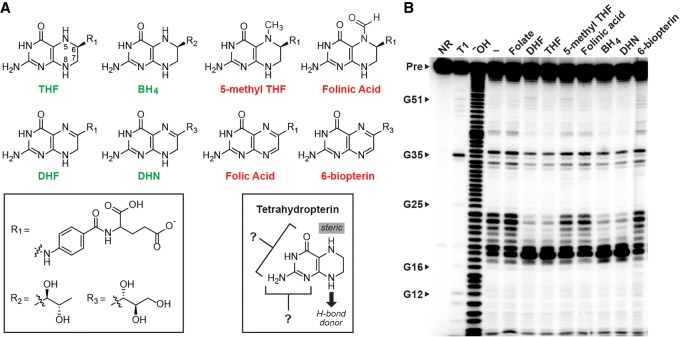
Molecular recognition by a *folE* motif RNA requires a reduced pterin moiety. (*A*) Chemical structures of ligands evaluated for binding by the *M. loti* 62 *folE* RNA. Molecule names (see legend to Fig. 1A for abbreviation descriptions) are colored green if they are bound by the RNA, and red if they are rejected. Based on the data in *B*, the variable groups (*left* box) are not involved in molecular recognition, whereas the chemical states of the atoms at positions 5 and 8 are critical (*right* box). (*B*) PAGE analysis of in-line probing assays with the *M. loti* 62 *folE* RNA construct with THF and its various chemical derivatives. Additional annotations are as described for Figure 2B.

Various analogs of THF (Fig. 4A) were used to explore the ligand specificity of the *M. loti* 62 *folE* RNA, also by using in-line probing assays (Fig. 4B). In addition to THF (*K*_D_ ∼ 19 µM, Fig. 2C), the compounds tetrahydrobiopterin (BH_4_), dihydrofolate (DHF) and 7,8-dihydroneopterin (DHN) are bound by the aptamer with near equal *K*_D_ values (∼17, 27, 18 µM, respectively). In contrast, no structural modulation was observed when the RNA was incubated with folic acid, 6-biopterin, 5-methyl THF or folinic acid, even at concentrations as high as 300 µM (Fig. 4B).

The ability of *folE* motif RNAs to discriminate against 5-methyl THF and folinic acid, yet be indifferent to the oxidation state of the N5 position of the pterin, suggests that the aptamer uses a steric block to discriminate against compounds that carry chemical moieties larger than a proton at this position (Fig. 4A, right box). The aptamer also fails to bind compounds such as folic acid and 6-biopterin, wherein N8 is in its oxidized form. This discrimination could be achieved if the aptamer forms a hydrogen bond with the ligand. However, we cannot rule out the possibility that the aptamer recognizes a difference in the pucker of the ring system that occurs with the change in redox states (Poe and Hoogsteen 1978). Finally, the type of chemical moiety attached at the C6 position has no effect on ligand binding affinity, indicating that the pterin moiety alone supplies the functional groups involved in ligand recognition.

Additional analogs would need to be examined to establish the importance of other functional groups of the pterin ring system. Unfortunately, some natural derivatives are relatively unstable and therefore not available for examination. However, the current findings are sufficient to conclude that the binding characteristics of the *folE* motif are distinct from a widespread riboswitch class for THF that was previously reported (Ames et al. 2010). For example, various natural folate derivatives that carry single carbon units (e.g., 5-methyl THF and folinic acid) are tightly bound by representatives of the THF riboswitch class (Ames et al. 2010; Trausch et al. 2011), but these same compounds are rejected by the *folE* motif aptamer. Furthermore, an atomic-resolution structural model for a THF-I riboswitch reveals the presence of two ligand binding sites, which was shown to bind two ligands cooperatively. Both binding sites have the space to accommodate bulky modifications to the N5 atom, whereas the *folE* motif RNA appears to form a steric block to such moieties.

Given the ability of the *M. loti* 62 *folE* RNA to broadly bind ligands with a partially or fully reduced pterin ring, the precise biologically relevant ligand sensed by this riboswitch class remains unclear. Representatives are always immediately adjacent to *folE* genes, which code for the enzyme that catalyzes the first committed step in folate biosynthesis. Thus, it might be possible that these riboswitches do not discriminate between THF, DHF, BH_4_, or DHN, but rather read out the concentration of the pool of molecules carrying the appropriate pterin ring forms and turn off *folE* gene expression when this pool is abundant. Alternatively, perhaps a single, more abundant folate derivative is sensed as a surrogate for the adequate production of the other similar compounds.

### Concluding remarks

Our findings support the hypothesis that *folE* motif RNAs function as members of a novel THF riboswitch class, which we propose naming “THF-II.” This second class of THF-sensing riboswitches is present in Gram-negative Rhizobiales species, whereas members of the previously reported class (Ames et al. 2010), which we propose renaming as “THF-I,” are more widely distributed among several Gram-positive bacterial divisions. Another prominent difference between the two riboswitch classes are their consensus sequence and structural models. The secondary structure model for the THF-II aptamer exhibits relatively simple architecture compared to most other riboswitch classes, which is very distinct from the secondary structure of THF-I riboswitch aptamers.

In addition to the difference in their phylogenetic distribution, the newly described THF-II riboswitch class is distinguished from THF-I aptamers through gene association, ligand binding characteristics, and aptamer architecture. For example, THF-II RNAs are exclusively located upstream of *folE* genes, whereas THF-I riboswitches are associated with a wider set of folate biosynthesis genes. Also, THF-I aptamers bind ligands carrying a tetrahydropterin core, but accept a variety of single-carbon moieties at the N5 position. In contrast, THF-II aptamers appear to recognize a wider range of THF analogs that contain reduced pterin moieties, but appear not to tolerate substituents at the N5 position beyond a hydrogen. Our binding data suggests that THF-II aptamers likely form a 1:1 complex with the ligand. In contrast, two binding sites are formed by at least some THF-I aptamers and these function cooperatively, although some also form a 1:1 interaction with their target ligand (Trausch et al. 2011). The existence of THF-II riboswitches highlights the ability of bacteria to perform similar biological functions using diverse RNA architectures that are tuned to the needs of the host organism.

One unusual feature of the in-line probing data for both the *M. loti* and *O. intermedium* 62 *folE* RNAs hints at a possible role for a highly conserved U residue. Although most internucleotide linkages that undergo scission rate changes are suppressed upon ligand binding, the U at position 18 of *M. loti* 62 *folE* experiences a substantial increase in the extent of spontaneous breakdown (Fig. 2B). A similar effect is observed for the *O. intermedium* 62 *folE* RNA at the equivalent position (Supplemental Fig. S1). This substantial ligand-dependent increase in RNA strand scission, coupled with the fact that the U nucleotide at this position is highly conserved, suggests that this position is involved in forming a tertiary structure that holds the phosphodiester linkage in an in-line geometry (Soukup and Breaker 1999). This would explain why nucleophilic attack of the 2′-oxygen atom of U18 on the adjacent phosphorus center would be best promoted by the RNA structure in its ligand-bound state.

Curiously, the M2 version of the *M. loti* 62 *folE* RNA exhibits a similarly robust cleavage product at U18 resulting from in-line probing, even when ligand is absent (Fig. 3). This result suggests that the C22G mutation creates a structure for the M2 construct that looks remarkably similar to the wild-type RNA construct despite the lack of a bound ligand. One possible explanation for this structural correspondence is that the C-to-G change at position 22 presents some of the same chemical structure as does the pterin ring of the natural ligand. It is known that THF-I riboswitch aptamers use U nucleotides to make direct contacts with their ligands (Trausch et al. 2011). Perhaps members of the THF-II riboswitch class use a U nucleotide in a structure that constrains its linkage in an in-line geometry when ligand binds, or when the G mutation at position 22 simulates the ligand.

The original simplicity of the secondary structure model and the limited bioinformatics data first observed for the *folE* motif (Weinberg et al. 2017) had initially dampened our enthusiasm for pursuing this as a riboswitch candidate. However, we recently validated several rare riboswitch classes with relatively small and simple architectures (Sherlock et al. 2017; Mirihana Arachchilage et al. 2018; Atilho et al. 2019), which elevated our interest in considering otherwise marginal orphan riboswitch candidates. In addition, the intriguing gene association for *folE* motif RNAs, coupled with the fact that the most abundant collection of riboswitch ligands are enzyme cofactors, prompted us to pursue this candidate for experimental validation. Another critical factor in our decision was the expanded bioinformatics data on *folE* motif RNAs, which revealed the consensus for the entire aptamer and provided a larger number of gene annotations. A similar outcome occurred in the effort to examine the rare SAM-VI riboswitch class (Mirihana Arachchilage et al. 2018). The continued discovery of rare, and sometimes small riboswitches is consistent with our prediction (Ames and Breaker 2010; McCown et al. 2017) that many thousands of classes remain to be found in diverse species of bacteria. The opportunity to make additional discoveries is expected to improve as DNA sequence databases expand with newly sequenced genomes.

Finally, evidence for the existence of the THF-II riboswitch class adds support for the view that similar RNA structures might have existed in the RNA World. THF and its various natural derivatives, along with other nucleotide-derived coenzymes, have been proposed to predate the emergence of proteins (Woese 1967; White 1976; Benner et al. 1989). This, coupled with the importance of regulating the production of essential enzyme cofactors, perhaps explains why more than a third of all known riboswitch classes sense and respond to these nucleotide derivatives (McCown et al. 2017; Mirihana Arachchilage et al. 2018) or their precursors (Atilho et al. 2019).

## MATERIALS AND METHODS

### Bioinformatics analysis

Additional *folE* riboswitch candidates were identified by using comparative sequence analysis algorithms CMfinder (Yao et al. 2006) and Infernal 1.1 (Nawrocki and Eddy 2013) as described previously (Weinberg et al. 2017). Genomic DNA databases examined included RefSeq version 80 and additional microbial environmental sequence collections. Secondary structure models were prepared using the software program R2R (Weinberg and Breaker 2011).

### Chemical and oligonucleotides

All chemicals and synthetic oligonucleotides were purchased from Sigma-Aldrich, with the exception of [γ-^32^P] ATP, which was purchased from PerkinElmer. Enzymes were purchased from New England BioLabs unless otherwise noted. A list of oligonucleotides used in this study can be found in Supplemental Table S1.

### RNA oligonucleotide preparation

Synthetic DNA oligonucleotides of the desired template sequences that contain a T7 RNA polymerase (T7 RNAP) promoter on the 5′ terminus were transcribed in vitro for 4 h at 37°C in a 100 µL reaction containing laboratory-prepared T7 RNAP (2 units µL^−1^), 80 mM HEPES-KOH (pH 7.5 at 23°C), 24 mM MgCl_2_, 2 mM spermidine, 40 mM DTT, and 2 mM of each of the four NTPs. The desired RNA product was isolated via denaturing (8 M urea) 10% polyacrylamide gel electrophoresis (PAGE, National Diagnostics). The gel band containing the desired RNA product was excised after UV shadowing using a hand-held UV lamp and a fluorescent TLC plate. RNA was extracted by crush-soaking for 30 min at 23°C in a 450 µL solution containing 200 mM NaCl, 10 mM Tris-HCl (pH 7.5 at 23°C), and 1 mM EDTA. RNA was precipitated from the resulting supernatant by the addition of 1 mL of ice-cold 100% ethanol, then pelleting via centrifugation at 13,000 rpm for 20 min. The resulting RNA pellet was dried under vacuum centrifugation, then resuspended in 20 µL deionized water (dH_2_O).

To generate 5′ ^32^P-labeled RNAs, 20 pmol of the RNA was dephosphorylated using rAPid alkaline phosphatase (Roche Life Sciences) according to the manufacturer's protocol. Half of this reaction was then ^32^P-radiolabeled at the 5′ terminus using T4 polynucleotide kinase in a 20 µL reaction mixture containing 25 mM CHES (pH 9.0 at 23°C), 5 mM MgCl_2_, 3 mM DTT and 20 µCi[γ-^32^P]ATP via incubation for 1 h at 37°C. The resulting radiolabeled RNA was purified by denaturing 10% PAGE and the desired RNA was extracted as described above.

### In-line probing assays

In-line probing experiments were conducted as previously described (Soukup and Breaker 1999; Regulski and Breaker 2008). Briefly, a trace amount (∼5 nM) of 5′ ^32^P-labeled RNA was incubated at 25°C in a 10 µL reaction volume for ∼40 h in the presence of 100 mM KCl, 50 mM Tris-HCl (pH 8.3 at 23°C), 20 mM MgCl_2_ and the presence or absence of the desired ligand. The RNA spontaneous cleavage products were resolved by denaturing 10% PAGE, and the resulting gel was dried and imaged with a PhosphorImager (GE Healthcare Life Sciences). Fraction bound values were established by varying the concentration of ligands in individual in-line probing reactions, quantifying regions that exhibited structural modulation using ImageQuant 5.1 (GE Healthcare Life Sciences), then normalized to a nonmodulating band, and finally scaled between 0 and 1. Apparent dissociation constant (*K*_D_) values were estimated by plotting fraction bound to the logarithm of the molar concentration of the ligand then overlaying a theoretical 1:1 binding curve (Hill coefficient of 1) on the data points using GraphPad Prism 7.

## SUPPLEMENTAL MATERIAL

Supplemental material is available for this article.

## Supplementary Material

Supplemental Material
