# Supplementary material for: Biochemical validation of a second class of tetrahydrofolate riboswitches in bacteria
Source: RNA. 2019 Sep;25(9):1091–7. doi: 10.1261/rna.071829.119 (PMC6800512; doi:10.1261/rna.071829.119)
Supplement: Supplemental Material [file supp_25_9_1091__index.html]

Biochemical validation of a second class of tetrahydrofolate riboswitches in bacteria — Supplemental Material 

# Biochemical validation of a second class of tetrahydrofolate riboswitches in bacteria

## Supplemental Material

- Supplemental\_Data.zip
- Supplemental\_Information.pdf
