# Supplementary material for: Biochemical validation of a second class of tetrahydrofolate riboswitches in bacteria
Source: RNA. 2019 Sep;25(9):1091–7. doi: 10.1261/rna.071829.119 (PMC6800512; doi:10.1261/rna.071829.119)
Supplement: Supplemental Material [file supp_071829.119_Supplemental_Information.pdf]

Supplemental Information

Biochemical validation of a second class of tetrahydrofolate riboswitches in bacteria

XI CHEN,[1,4] GAYAN MIRIHANA ARACHCHILAGE,[2,4] AND RONALD R. BREAKER[1,2,3]

[1]Department of Molecular Biophysics and Biochemistry, Yale University, New Haven, Connecticut 06520-8103, USA
[2]Howard Hughes Medical Institute, Yale University, New Haven, Connecticut 06520-8103, USA
[3]Department of Molecular, Cellular and Developmental Biology, Yale University, New Haven, Connecticut 06520-8103, USA

[4]These authors contributed equally to this work.

Corresponding author: ronald.breaker@yale.edu

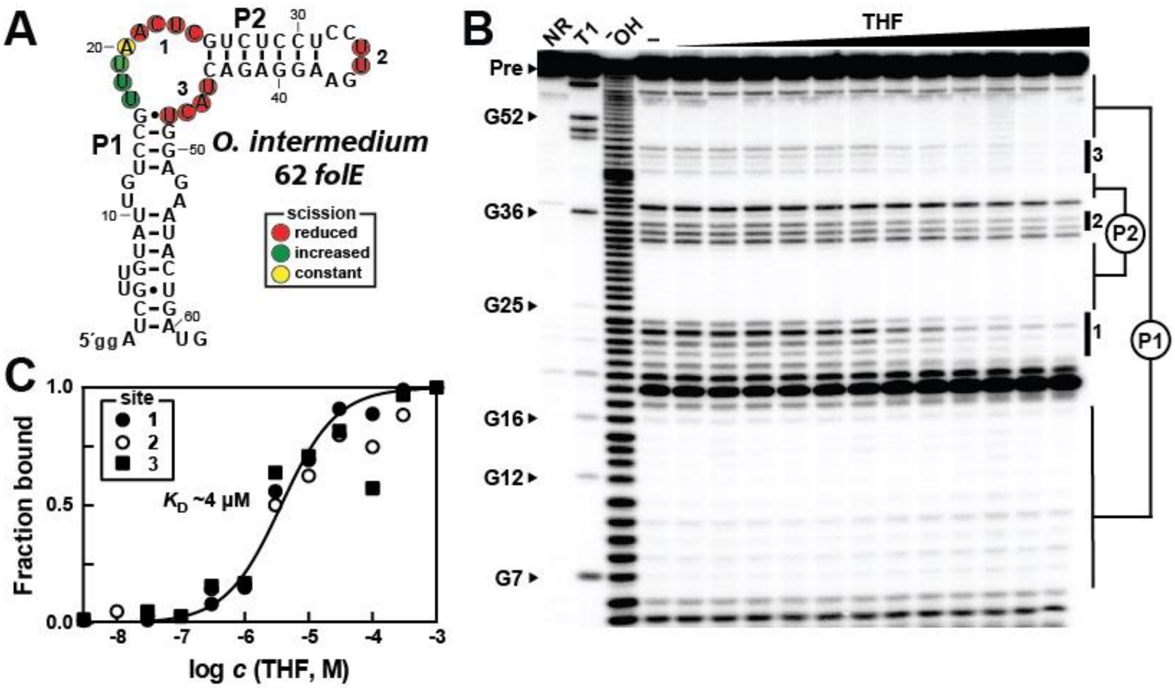

FIGURE S1. THF binding by the 62 *folE* RNA construct derived from *Ochrobactrum intermedium*. (*A*) Sequence and secondary structure model of the *O. intermedium* 62 *folE* RNA construct. Additional notations are as described in the legend to **Fig. 2A.** (*B*) PAGE analysis of the *O. intermedium* 62 *folE* RNA subjected to in-line probing assays in the absence (–) or presence of THF concentrations ranging from 10 nM to 1 mM. Additional annotations are as described in the legend to **Fig. 2B.** (*C*) Plot of fraction of RNA bound to THF versus the logarithm of the molar concentration of THF. The data is derived from B. Additional annotations are as described in the legend to **Fig. 2C**.

Supplemental Table S1. Sequences of synthetic DNAs used in this study.

Name	Sequence (5′ to 3′)	Annotation
folE1	CATAAAGGCGTCTCCCGTA GTTCCTCTCGAGGAGGAAC GAGTTGAACGGACCACGA CCTTTCCTATAGTGAGTCG TATTA	The template for transcription of *M. loti 62 folE* RNA
folE2	CATAAAGGCGTCTCCCGTA GTTCCTCTCGAGGAGGAAC GAGTTGTACGGACCACGAC CTTTCCTATAGTGAGTCGT ATTA	The template for transcription of M1 *M. loti 62 folE* RNA
folE3	CATAAAGGCGTCTCCCGTA GTTCCTCTCGAGGAGGAAC GACTTGAACGGACCACGAC CTTTCCTATAGTGAGTCGT ATTA	The template for transcription of M2 *M. loti 62 folE* RNA
folE4	CATAAAGGCGTCTCCCGCA GTTCCTCTCGAGGAGGAAC GAGTTGAACGGACCACGA CCTTTCCTATAGTGAGTCG TATTA	The template for transcription of M3 *M. loti 62 folE* RNA
folE5	CATCAGTATTCTCCAGTAG TCTCCTTCAAGGAGGAGAC GAGTTAAACGGACAATAC AACGATCCTATAGTGAGTC GTATTA	The template for transcription of *O. intermedium 62 folE* RNA
